# Cancer vaccines: shared tumor antigens return to the spotlight

**DOI:** 10.1038/s41392-020-00364-8

**Published:** 2020-10-30

**Authors:** Lijin Li, S. Peter Goedegebuure, William Gillanders

**Affiliations:** 1 Department of Surgery, Washington University School of Medicine, St Louis, MO USA; 2 The Alvin J. Siteman Cancer Center at Barnes-Jewish Hospital and Washington University School of Medicine, St Louis, MO USA

**Keywords:** Cancer therapy, Tumour immunology

Sahin et al. recently reported interim data from a phase 1 clinical trial (NCT02410733) treating melanoma patients with a RNA-lipoplex vaccine (RNA-LPX), targeting melanoma TAA.^1^ The trial is a multicenter, non-randomized, open-label, dose-escalation trial designed to evaluate the safety and tolerability of BNT111 targeting four melanoma TAA (NY-ESO-1, MAGE-A3, tyrosinase, and TPTE). Secondary endpoints include vaccine-induced immune responses and clinical response of patients with measurable disease.

Cancer vaccines have a mixed track record but the recent marked success of immune checkpoint blockade (ICB) immunotherapy has renewed interest in cancer vaccines. Cancer vaccine targets include TAA and more recently, cancer neoantigens. TAA include proteins that are aberrantly expressed in tumors compared with normal tissues (overexpression, different subcellular localization), or proteins that are expressed in tumors but are normally sequestered in immune-privileged sites, or produced only in certain differentiation stages. The prevalence of TAA in cancers makes it possible to design and manufacture off-the-shelf vaccines targeting TAA.

Unfortunately, there are challenges targeting TAA, and cancer vaccines targeting TAA have proven to be disappointing. TAA vaccines must overcome central and acquired tolerance, limiting the magnitude of induced T-cell responses. Many vaccine platforms have proven to be suboptimal, inducing weak and/or short-lived immune responses.^2^ Finally, TAA vaccines may result in autoimmunity. Such off-tumor, on-target toxicity, although tolerable, has been observed in clinical studies. It remains unclear if the disappointing results of studies targeting TAA is a result of the inability to induce robust immune responses, or the inability (until recently) to effectively target immune checkpoints.

**Fig. 1:**
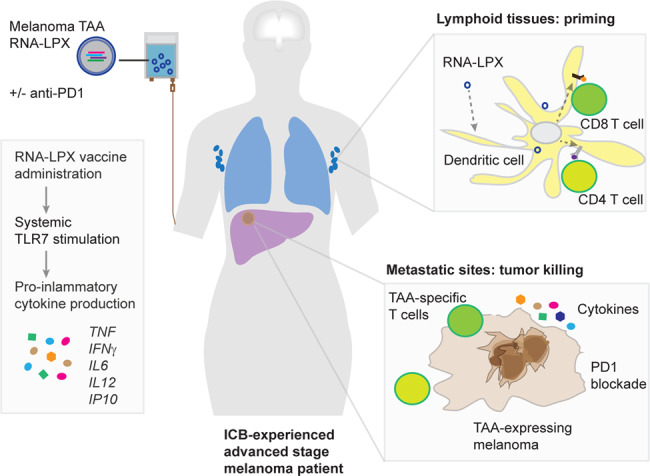
Melanoma RNA-LPX vaccine targeting TAA induces antitumor immunity in ICB-experienced patients. The lipid-formulated vaccine is composed of RNA encoding multiple TAAs. RNA vaccine stimulates TLR7, which results in systemically increased production of pro-inflammatory cytokines. RNA-LPX are taken up by DCs and the antigens are presented to T cells in the context of HLA class I and class II molecules. Combined with PD1 blockade, the off-shelf TAA-targeting RNA vaccine can lead to favorable clinical outcomes. Further studies are required to determine the optimal strategy for integrating cancer vaccines into clinical treatment paradigms

The trial involved 89 patients with advanced melanoma that express at least one of the four targeted TAA. Patients were treated with repeated doses of intravenously delivered BNT111 ranging from 7.2 to 400 µg. Some cohorts received vaccine alone, whereas others were also treated in combination with anti-PD1 therapy. Of note, almost all patients had received prior ICB therapy (anti-CTLA4, anti-PD1, or both). Most patients experienced mild to moderate, transient flu-like symptoms including pyrexia (82%) and chills (71%). The investigators also documented a transient upregulation of plasma cytokines (IFNα, IFNγ, IP10, IL6, and IL12) accompanied by a transient increase of body temperature, indicating an inflammatory response induced by RNA interaction with toll-like receptors (TLR). This is consistent with the RNA-LPX vaccine’s proposed mechanism of action (Fig. 1) inducing a transient cytokine response and targeting immature DC, resulting in a Th1-skewed T-cell response.^3^

Ex vivo IFNγ ELISpot assays were performed on 50 patients before and after vaccination. In all, 39/50 (78%) showed immune responses against at least one TAA, indicating a robust response to the vaccine. Most of the responses were CD8^+^ T-cell responses, with de novo responses being more common than expansion of pre-existing responses. Studies were also performed after in vitro stimulation in 20 patients. All 20 patients tested responded to at least one TAA with the majority being CD4 responses alone, or both CD8 and CD4 responses. In these assays, CD8^+^ T cells showed an effector memory phenotype and produced both TNF and IFNγ. T-cell responses induced by the RNA-LPX seemed to be independent of whether or not the vaccine treatment was combined with anti-PD1 ICB.

Clinical activity was evaluated in a subset of 42 ICB-experienced patients with measurable disease. Twenty-five patients received the vaccine as a single agent. Three experienced a partial response, and seven showed stable disease. Seventeen patients were treated with the vaccine in combination with anti-PD1. Six of them developed a partial response (35%), a response rate similar to response rates for PD1 blockade alone in ICB-naïve melanoma patients. There was a correlation between T-cell responses and favorable clinical outcomes.

It is notable that the RNA-LPX vaccines are able to induce robust immune responses to TAA. The RNA-LPX platform is formulated as a liposome to protect RNA from degradation and target immature DCs in lymphoid tissues.^3^ RNA also activates the TLR7 signaling pathway and induces an inflammatory cytokine response, driving DC maturation, and the presentation of TAA by both MHC class I and class II molecules. The ability to induce robust immune responses to TAA suggest that RNA-LPX vaccines represent an attractive delivery platform to target TAA. Other vaccine platforms are currently under active investigation (peptide, DNA, viral, and cellular vaccines). It remains unclear if the low rate of clinical benefit is related to the limited ability of many vaccine platforms to induce robust responses or other factors such as the inability to overcome immune checkpoints and/or regulatory networks that restrain immune responses in the tumor microenvironment.^2^

There is currently great interest in combining ICB with other immune therapies including cancer vaccines. It remains unclear how to best combine these therapies. The RNA-LPX vaccine study is notable given that some patients who failed previous ICB experienced a clinical response to the RNA-LPX vaccine. A recent study by Verma et al. highlights some of the challenges of trying to combine ICB with cancer vaccines.^4^ The investigators demonstrated that PD1 blockade of suboptimally primed CD8^+^ T cells (as typically seen in cancers) results in dysfunctional T cells characterized by upregulation of CD38, and the inability to respond to subsequent antigen or vaccine stimulation. This suggests that cancer vaccine administration following failed ICB may not be ideal. The RNA-LPX vaccine study shows that it may be possible to reverse this anti-PD1-induced CD8^+^ T-cell dysfunction under certain conditions. Additional study will be necessary to determine the best strategies to combine ICB and cancer vaccines.

Overall, the results of the RNA-LPX vaccine targeting melanoma TAA are encouraging. Success targeting melanoma TAA has important implications beyond melanoma given that promising TAA have been identified in other cancer types. In addition to TAA, RNA-LPX, peptide, and DNA vaccines targeting cancer neoantigens are being tested. Unlike TAA, neoantigens are derived from genetic alterations and are tumor specific. Targeting neoantigens requires a personalized approach with minimal risk of collateral damage. Neoantigens are also not subject to central tolerance and can trigger robust immune responses. Early results of neoantigen cancer vaccines are promising but limited clinical data make it difficult to meaningfully compare neoantigen vaccines to TAA vaccines. Vaccines targeting both TAA and cancer neoantigens may prove more effective than targeting either TAA or neoantigens alone.^5^ The present study highlights the potential of cancer vaccines leveraging robust vaccine platforms in combination with ICB. Given the safety profile of cancer vaccines, additional study should be prioritized to best define strategies to integrate cancer vaccines into existing clinical treatment paradigms. Given recent insights into the biology of immune checkpoints and the tumor microenvironment, cancer vaccines will likely need to be combined with other immune therapies to reach full potential.
